# Serological detection of Tick-Borne Relapsing Fever in Texan domestic dogs

**DOI:** 10.1371/journal.pone.0189786

**Published:** 2017-12-12

**Authors:** Maria D. Esteve-Gasent, Chloe B. Snell, Shakirat A. Adetunji, Julie Piccione

**Affiliations:** 1 Department of Veterinary Pathobiology, College of Veterinary Medicine and Biomedical Sciences, Texas A&M University, College Station, Texas, United States of America; 2 Texas A&M Veterinary Medical Diagnostic Laboratory, College Station, Texas, United States of America; University of Maryland, College Park, UNITED STATES

## Abstract

Tick-Borne Relapsing Fever (TBRF) is caused by spirochetes in the genus *Borrelia*. Very limited information exists on the incidence of this disease in humans and domestic dogs in the United States. The main objective of this study is to evaluate exposure of dogs to *Borrelia turicatae*, a causative agent of TBRF, in Texas. To this end, 878 canine serum samples were submitted to Texas A&M Veterinary Medical Diagnostic Laboratory from October 2011 to September 2012 for suspected tick-borne illnesses. The recombinant Borrelial antigen glycerophosphodiester phosphodiesterase (GlpQ) was expressed, purified, and used as a diagnostic antigen in both ELISA assays and Immunoblot analysis. Unfortunately, due to significant background reaction, the use of GlpQ as a diagnostic marker in the ELISA assay was not effective in discriminating dogs exposed to *B*. *turicatae*. Nevertheless, immunoblot assays showed that 17 out of 853 samples tested were considered to be seropositive, which constitutes 1.99% of all Texas samples tested in this study. The majority of positive samples were from central and southern Texas. Exposure to TBRF spirochetes may be seasonal, with 70.59% (12 out of 17) of the cases detected between June and December. In addition, 2 out of the 17 sero-reactive cases (11.76%) showed reactivity to both *B*. *burgdorferi* (causative agent of Lyme disease) and *B*. *turicatae* (a causative agent of TBRF). This is the first report of TBRF sero-prevalence in companion animals in an endemic area. Our findings further indicate that *B*. *turicatae* is maintained in domestic canids in Texas in regions where human disease also occurs, suggesting that domestic dogs could serve as sentinels for this disease.

## Introduction

Tick-Borne Relapsing Fever (TBRF) is a zoonotic disease caused by certain species of *Borrelia*, including *Borrelia hermsii*, *B*. *parkerii* and *B*. *turicatae* in the U.S. [1, 2]; and *B*. *persica*, *B*. *hispanica*, *B*. *duttoni*, *B*. *coriaceae* and *B*. *crocidurate* in Southern Europe and African countries [1]. TBRF affects both humans and companion animals, particularly dogs [2, 3]. These pathogens are transmitted by soft-bodied tick of the genus *Ornithodoros*. For example, *O*. *hermsii* ticks transmit *B*. *hermsii* in western U.S., while *O*. *turicata* ticks transmit *B*. *turicatae* in southern U.S. [4, 5]. In comparison to hard ticks, soft-bodied ticks can feed for only a short period of time, sometimes as little as a few minutes [4, 6, 7]. Previous studies by Lopez et. al. have documented that *B*. *turicatae* can be transmitted to hosts through the bite of *O*. *turicata* in as short as 15 seconds [7]. This is possible due to the fact that *B*. *turicatae* resides in the salivary glands of *O*. *turicata* allowing such rapid transmission [8]. In addition, *O*. *turicata* ticks have a nocturnal feeding behavior [9, 10] and are often not noticed by affected people, pet owners, and veterinarians [4, 11, 12]. Nevertheless, when diagnosed in a timely manner, TBRF appears to be treatable [2]. The most common clinical signs in dogs are fever, lethargy, joint pain, neurologic signs, organ damage, and in some cases death [3, 13–15].

Although primarily reported in dogs, TBRF has also been reported in horses, bats, and cats [1, 3, 14–16]. Since the 1970’s a few dogs throughout the United States have been confirmed to be infected with TBRF *Borrelia* [3, 13, 14, 16]. Despite these confirmed cases, no major studies have been performed to determine the prevalence or the distribution of the TBRF in dogs in the U. S. [3, 13]. In a recent study by our group, 5 canine TBRF cases were described [3] in Texas, with this diagnosis confirmed by microscopy and PCR. Currently, a reliable and cost effective high throughput diagnostic test does not exist for the identification of TBRF despite the fact that immunologically distinct antigens such as rGlpQ [17–19] and BipA [20, 21] have been identified and utilized in people. Consequently, the only diagnostic method currently employed by pathologists and practitioners for the diagnosis of TBRF both in human and veterinary medicine is the presence of spirochetes in a blood smear (gold standard) and the detection of moderate to severe thrombocytopenia with or without anemia in febrile patients [22, 23].

In the U.S. there have been a total of 504 human TBRF cases reported to the CDC from 1990 to 2011 [2]. All the cases were acquired in 12 states (Arizona, California, Colorado, Idaho, Montana, Nevada, New Mexico, North Dakota, Oregon, Texas, Utah and Washington). Of those states, California, Washington and Colorado reported 70% of all cases [2, 24]. On average a total of 20 cases per year were reported to CDC. In addition, the majority of the cases were reported during the summer months (June through September) with a peak in July and August. Interestingly, human cases in Texas were mostly reported in the fall months, and extending through the winter and spring. In previous epidemiological studies, it has been reported that the disease affects mostly males (57%), with a median age of 38 years (ranging from 1 to 91 years old), with a bimodal age distribution where most of the cases correspond to 2 age groups, 10–14 years and 40 to 44 years [2, 9]. TBRF is not a nationally reportable disease in the U.S., and thus, a standard case definition is not available, and important epidemiological information is missing [2, 24]. In addition, a recent study has evaluated the distribution of *Ornithodoros turicata* and potential vertebrate hosts in Southern US proposing distribution models that extend through most of the state of Texas and regions in Oklahoma and New Mexico, extending further South into the transboundary region with Mexico [5].

Due to various reports of canine [3, 14] and human [11, 25] TBRF cases in Texas, and a recent study on the geographic distribution of the tick vector *O*. *turicata* [5], the aim of this study was to evaluate the presence of sero-reactive dogs to the TBRF spirochete *B*. *turicatae* in the state of Texas, and to evaluate both, its geographic and temporal distribution. A total of 878 canine serum samples collected from October 2011 through September 2012 were tested. Hence, the present study is the first longitudinal study performed in one of the TBRF endemic states with the objective of further characterizing this disease in dogs. Finally, the potential role of dogs as sentinels for TBRF is suggested.

## Materials & methods

### Ethics

A total of 878 canine serum samples were transferred from the Texas A&M Veterinary Medical Diagnostic Laboratory (TVMDL) to the College of Veterinary Medicine & Biomedical Sciences at Texas A&M University after the 15-day legal hold period, in accordance with the Material Transfer Agreement between both institutions. No confidential information regarding the pet owners and/or veterinary clinic where the animals were evaluated was provided. The study was reviewed by Texas A&M University Animal Care and Use Committee (IACUC), since the samples were considered “Surplus Sera” from regular diagnostic services (samples with completed testing and ready for disposal), Texas A&M University IACUC decided the study was exempt of any permit. Therefore, no recruitment of animals specific to the current study was performed, and no direct handling of animals was done by the research team. The experiments described in here were conducted under the Institutional Biosafety Permit number 2010–036 and 2013–039.

### Canine serum samples

All serum samples were obtained from dogs suspected of having a tick-borne illness during October 2011 to September 2012. The information obtained from TVMDL for each sample included the case number, county of origin, zip code, the sample arrival date, the Lyme IFA analysis completion date, and the IFA test results. All samples were tested at TVMDL for Lyme Disease (LD) utilizing standardized IFA tests (Focus Diagnostics, Inc.). Dr. Job Lopez provided the four negative and one positive control sera used in this study. Samples obtained at different times of the year came from different animals (i.e., no repeated measurements were taken) [26].

### *Borrelia turicatae* cultures

In this study, the TCBP2 strain of *B*. *turicatae*, provided by Dr. T. Schwan at the Laboratory of Zoonotic Pathogens, Rocky Mountain Laboratories, NIAID, NIH, in Hamilton, MT, was used. This strain was previously isolated from a sick dog from Texas [14]. *B*. *turicatae* was grown in BSK-II media complemented with 12% inactivated rabbit serum. Cultures were incubated at 32°C and 1% CO_2_. When cultures reached a cell density of 5*10^7^ spirochetes/ml cells were harvested, washed 3 times with Hans Balance Salt Solution (HBSS, HyClone, ThermoFisher Scientific Inc., Waltham, MA), re-suspended in 100μl of HBSS and lysed by adding 100μl of Lauryl final sample buffer (FSB) so as to be ready for SDS-PAGE gel loading. Aliquots of *B*. *turicatae* lysates were stored at -20°C until use in SDS-PAGE gels electrophoresis.

### Recombinant GlpQ purification and concentration

**Fig 1:**
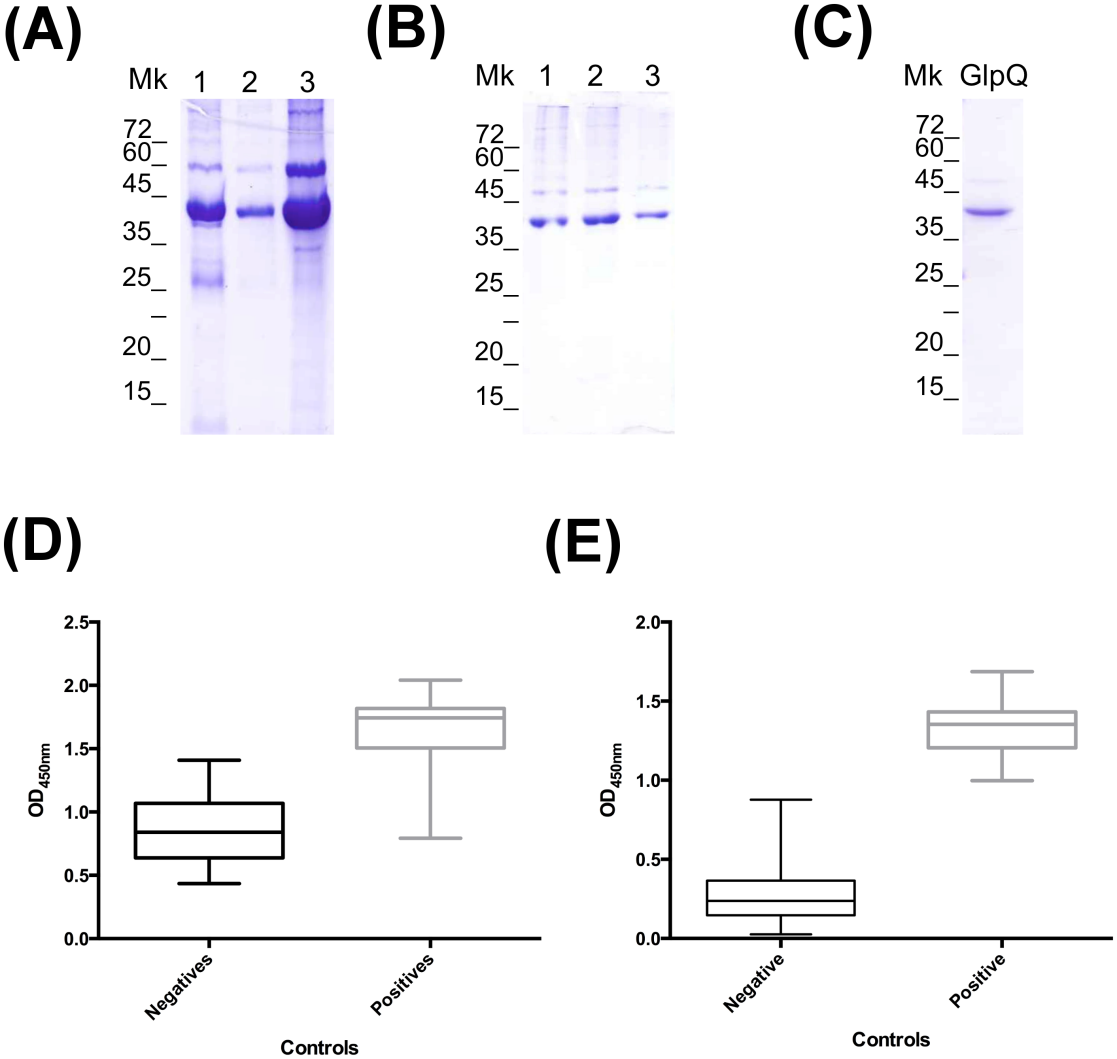
Purification and clean-up of rGlpQ for ELISA test use. (**A**) Optimization of enzyme concentration needed to cleave the fusion protein. rGlpQ was incubated with different concentrations of EKMax^™^ (lane 1: 0.1 Units/20μg rGLPQ, lane 2: 1 Units/20μg rGLPQ and lane 3: uncleaved rGlpQ) at 37°C overnight, and products were separated in 12% SDS-PAGE gels. (**B**) Standardization of the incubation time required for complete cleave of fusion protein using 0.5 units/20μg rGLPQ (lane 1: Uncleaved; lane 2: 17hrs and lane 3: 24hrs) at 37°C. (**C**) Purified rGlpQ after fusion protein removal and ready to use in ELISA and Immunoblot assays. (**D**) ELISA results of negative and positive controls using uncleaved rGlpQ and cleaved one (**E**). Mk: Molecular Marker used in SDS-PAGE gels.

The recombinant GlpQ (rGlpQ) was provided by Dr. Job Lopez and expressed as a fusion protein with His-thyrodoxin. The rGlpQ expressed as a fusion protein was causing non-specific antibody binding when used in the ELISA assay (Fig 1D). Therefore, the fusion protein thyrodoxin was removed. To this end, the rGlpQ, was incubated with three different concentrations of the enzyme EK-Max^™^ (Invitrogen Life Technologies, Carlsbald, CA): 1.0 units/20μg rGlpq, 0.5 units/20μg rGlpQ, and 0.1 units/20μg rGlpQ overnight at 37°C (Fig 1A). To standardize the enzyme incubation time, to obtain consistent cleaved rGlpQ, 0.5 units of enzyme per 20μg rGlpQ was incubated at 37°C for 17hr and 24hr (Fig 1B). The reactions were analyzed by running SDS-PAGE gels visualized using Coomassie blue staining. EK-Max^™^ was then removed by incubating the reaction mixture with EK-Away^™^ (Invitrogen Life Technologies, Carlsbald, CA) as per manufacturer’s protocol. To confirm that the cleaved rGlpQ was pure after the enzyme was removed, the final product was analyzed by SDS-PAGE gel (Fig 1C). Next, the cleaved rGlpQ antigen was concentrated using a 10 kDa centrifugal filter (Amicon^®^ EMD Millipore, Billerica, MA) and quantified using the BCA Protein Assay kit (Pierce^™^ ThermoFisher Scientific Inc., Waltham, MA) as per manufacturers’ protocols. Plates were read at a wavelength of 590nm using a plate reader and software (BMG LABTECH OMEGA, Germany).

### rGlpQ ELISA

Ninety-six well plates (Nunc ^®^ Thermo Scientific Ltd., Waltham, MA) were coated with 500ng/well of the cleaved rGlpQ for 2 hours at room temperature in coating buffer (pH 9.6). Plates were blocked overnight at 4°C with 3% Bovine Serum Albumin (BSA) in Phosphate Buffer Saline containing 0.5% Tween 20 (PBS-T). A 1:400 dilution of each canine sample was incubated in triplicates at room temperature for 1 hour. The plates were then incubated with a 1:3000 dilution of anti-dog HRP conjugated antibody (Rockland, Inc., Boyertown, PA) for 1 hour at room temperature. Plates were developed using *o*-phenylenediamine dihydrochloride substrate (Pierce^™^ ThermoFisher Scientific Inc., Waltham, MA) and then, the optical density at 450nm (OD_450nm_) was recorded using an ELISA plate reader (BMG LABTECH OMEGA, Ortenberg, Germany). Between each incubation step, plates were washed 3 times for 5 minutes each with PBS-T. All dilutions were made in PBS-T + 1% BSA.

### Immunoblot assays

#### *Borrelia turicatae* in-house assay

To confirm the ELISA seropositive samples, rGlpQ and *Borrelia turicatae* whole cell lysate were separated in 12% SDS-PAGE gels and wet transferred onto nitrocellulose membrane at 100 V for 2 hours. The membranes were blocked overnight at 4°C in 10% skin milk in Tris-Buffered Saline + 0.2% Tween-20 (TBS-T). After washing 3 times for 5 minute each using TBS-T, the membranes were incubated with 1:1000 dilutions of each canine serum sample for 1 hour at room temperature. The membranes were then washed again 3 times with TBS-T for 5 minutes each. Next, the membranes were incubated for 1 hour at room temperature with 1:3000 dilutions of anti-dog HRP conjugated antibody (Rockland, Inc., Boyertown, PA). After washing 6 times for 5 minutes each with TBS-T, the membranes were incubated with Chemiluminescent ECL detection reagents (GE Healcare, Piscataway, NJ) for 1 minute. Films were exposed to the membranes for 2 sec, 5 sec, 10 sec, 30 sec, 1 min, and 5 min and then developed. All dilutions were made in TBS-T plus 10% skin milk.

#### *Borrelia burgdorferi* commercially available assay

To confirm potential cross- reaction with *B*. *burgdorferi*, twenty-six dog serum samples were evaluated by Immunoblot using a commercially available human *B*. *burgdorferi* strip system (Trinity Biotech, *B*. *burgdorferi* Marblot^™^ Strip Test System, Ireland), adapted to test dog samples [26]. These 26 animals were suspected of a tick-borne illness, and had a positive ELISA for both *B*. *burgdorferi* [26] and *B*. *turicatae* (this study). For each sample or control strip (positive and negative), a channel in a 12-strip plate was filled with 2 ml of 1X sample diluent/wash solution provided by the manufacturer. After strips were equilibrated for 5 minutes, 20 μL of each of the samples (1:100) was added to the appropriately marked channel and incubated at room temperature for 30 minutes. Strips were washed three times by adding 2 mL of sample diluent/wash solution to each channel of the strip incubation tray and incubated for 5 minutes with vigorous agitation. Two ml of anti-dog alkaline phosphatase conjugated IgG antibody (Rockland Immunochemicals, Gilbertsville, PA) diluted 1:2,000 was added to each strip containing well, and incubated for 30 minutes at room temperature. Strips were then washed 3 times and 2 mL of color developing solution was added to each channel. All strips were incubated for 6 minutes to allow color development. Strips were then washed with 2 mL of deionized water, air-dried, and evaluated. The presence or absence of the following 13 bands was then recorded: a93, a66, a60, a58, a45, a41 (Flagella), a39 (BmpA), a34, a31 (OpsA), a30, a28, a23 (OpsC) and a18. All weak reactive bands were annotated as negative [26]. As previously described, bands a66, a58 and a45 were not considered due to their characteristic cross-reactivity. The presence of reactivity of two or more of the following antigens a93, a30, a 23 and/or a18 together with any of the other bands (excluding a66, a58 and a45) will be considered positive for LD [26].

### *Borrelia turicatae* specific Polymerase Chain Reaction (PCR)

The presence of *B*. *tuticatae* DNA in the dog serum samples was evaluated in specimens with positive results for both, TBRF and LD serological testing. A specific PCR to amplify *glpQ* was performed as previously described [15, 17]. This marker was used due to its specificity to TBRF *Borrelia* and complete absence in LD *Borrelia* sp [3]. Briefly, DNA was extracted from 100μl of dog sera using the High Pure PCR Template Preparation kit (Roche Life Sciences, Inc. Indianapolis, IN) and following manufacturer’s recommendations. The DNA extraction and PCR protocol were carried out in different laboratories, and all PCR reactions were set up in a PCR workstation to avoid cross-contamination of specimens. In addition, a negative or water control and a positive control containing *B*. *turicatae* genomic DNA (provided by Dr. T. Schwan from Rocky Mountain Laboratories) were utilized in this study. PCR amplification was done using primers 128f (5’-CAG AAC ATA CCT TAG AAG CTC AAG C-3’) and 340r (5’-GTG ATT TGA TTT CTG CTA ATG TG-3’) previously described [17], and visualized by electrophoresis using 0.8% agarose gels and imaged using a ChemiDoc Touch^™^ (BioRad Laboratories, Inc. Hercules, CA).

### Statistics and cut off determination

To determine the cut off value to analyze the ELISA OD_450nm_ results we used the formula: (average of negative controls + (3 X Standard deviation of negative controls)). However, it was determined that while statistically significant, this value was too low since the negative controls used were not representative of the entire population. Because of this, and in order to avoid false positive samples, the cut off value was calculated as: (average of negative controls + (3 X upper 95% CI of the negative controls)).

## Results

### GlpQ antigen purification

In order to obtain a pure rGlpQ antigen to be used in both ELISA and Immunoblot assays, the thyrodoxin fusion was enzymatically removed. After analyzing the enzymatic reactions by SDS-PAGE gel it was found that the optimal concentration of EK-MaxTM enzyme and incubation time were 0.5 units/20μg rGlpQ and 24 hr respectively (Fig 1A and 1B). These conditions were used throughout the completion of this study.

### Sero-reactivity of canine serum samples by ELISA testing

**Fig 2:**
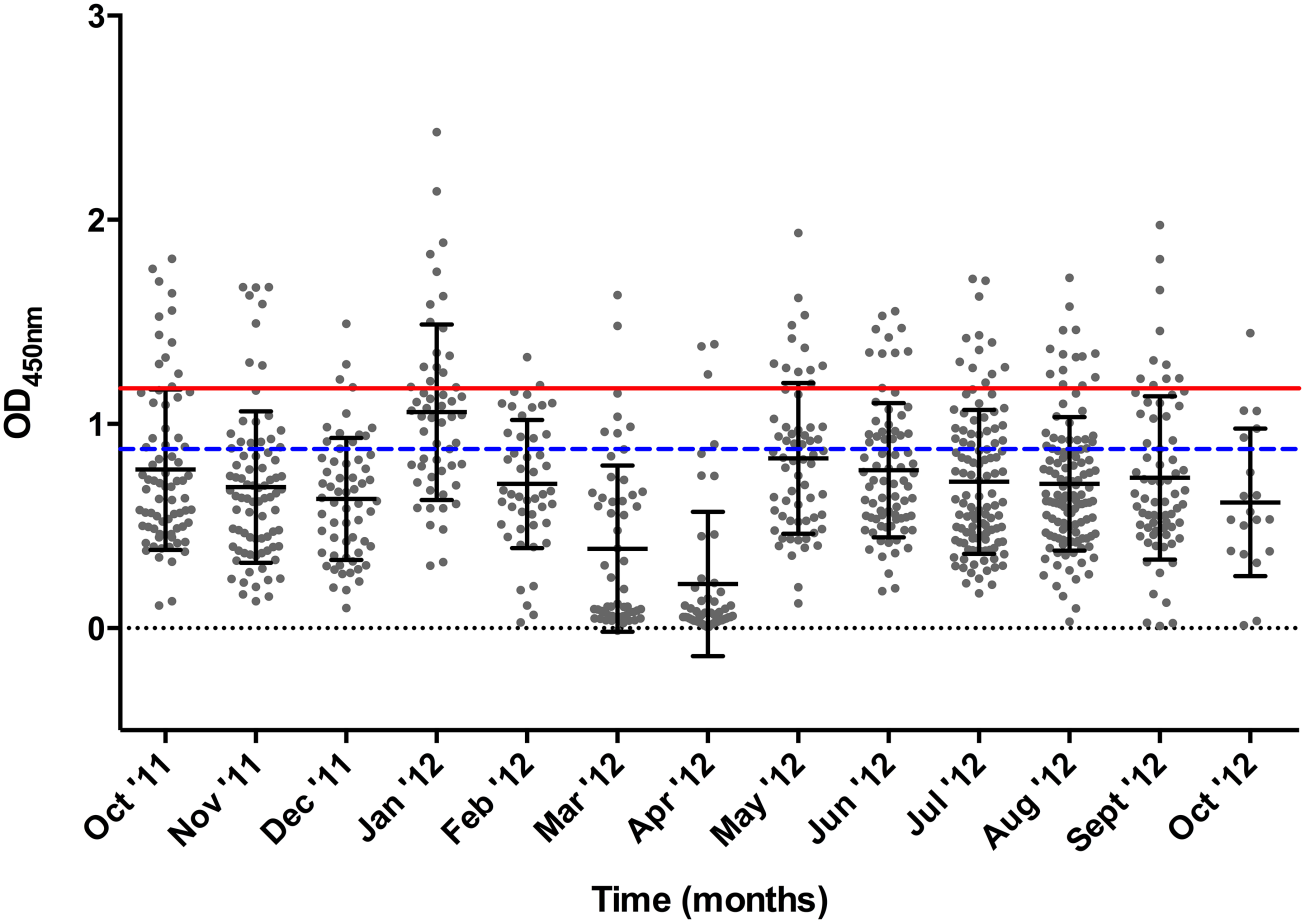
ELISA results of 878 canine serum samples tested in the study. The cleaved rGlpQ substrate was used in this assay. The red line denotes the cut off value used in the study. Cut off was established as the average of negative controls + (3 × upper 95% CI of the negative controls). The blue dashed line reference to a primary cut off value calculated as the average of negative controls + (3 × SD of negative controls).

By removing the thyrodoxin fusion protein the variation seen within the positive and negative controls read out was significantly reduced from OD_450nm_ range of 0.435–1.338 for the negative controls and 0.793–2.041 for the positive controls (Fig 1D) to 0.025–0.876 for the negative controls and 0.997–1.687 for the positive controls (Fig 1E). These results confirm the need to remove the thyrodoxin fusion protein from the rGlpQ antigen, allowing the determination of a cut off value for the ELISA test. By utilizing the formula (Average of negative controls + (3 X Standard deviation of negative controls)), the cut of value was set as 0.787 (Fig 2, blue dash line) giving a total of 340 sero-reactive samples. To provide a more stringent cut off value that will reduce the probability of false positives, we applied the formula (Average of negative controls + (3 X Upper 95% CI of the Negative controls)), and provided a cut off value of 1.191 (Fig 2, red line), with a total of 98 sero-reactive samples that will need to be confirmed by immunoblot assay.

Of the 98 sero-reactive specimens, 25 samples were from out of state and were not further analyzed in this study. The remaining 73 sero-reactive specimens from the state of Texas (Table 1) were further analyzed. As shown in Table 1, 72.61% of the sero-reactive samples were submitted from counties in the following eco-regions: East Central Texas plains, South Texas Plains, Texas Black Prairie and the Cross-Timbers.

**Table 1:** Percent of ELISA sero-reactive canine serum samples by eco-region in the state of Texas.

Eco-Region	Sero-reactive Samples*	Percentage (%)
East Central Texas Plains	21^⌘^	28.77
South Texas Plains	12^⌘^	16.44
Texas Black Prairie	10^⌘^	13.70
Cross-Timbers	10	13.70
Western Gulf Coastal Plains	9^⌘^	12.32
South Central	7	9.59
Others^	4	5.48
**Total**	**73**	**100%**

* Samples were considered sero-reactive by ELISA test using the rGlpQ antigen.

^⌘^ Eco-regions with cases positive by immunoblot.

^ Includes eco-regions with only 1 sero-reactive sample (High Plains, South Plains, Edwards Plateau and Central Great Plains).

### Western blot confirmation

**Fig 3:**
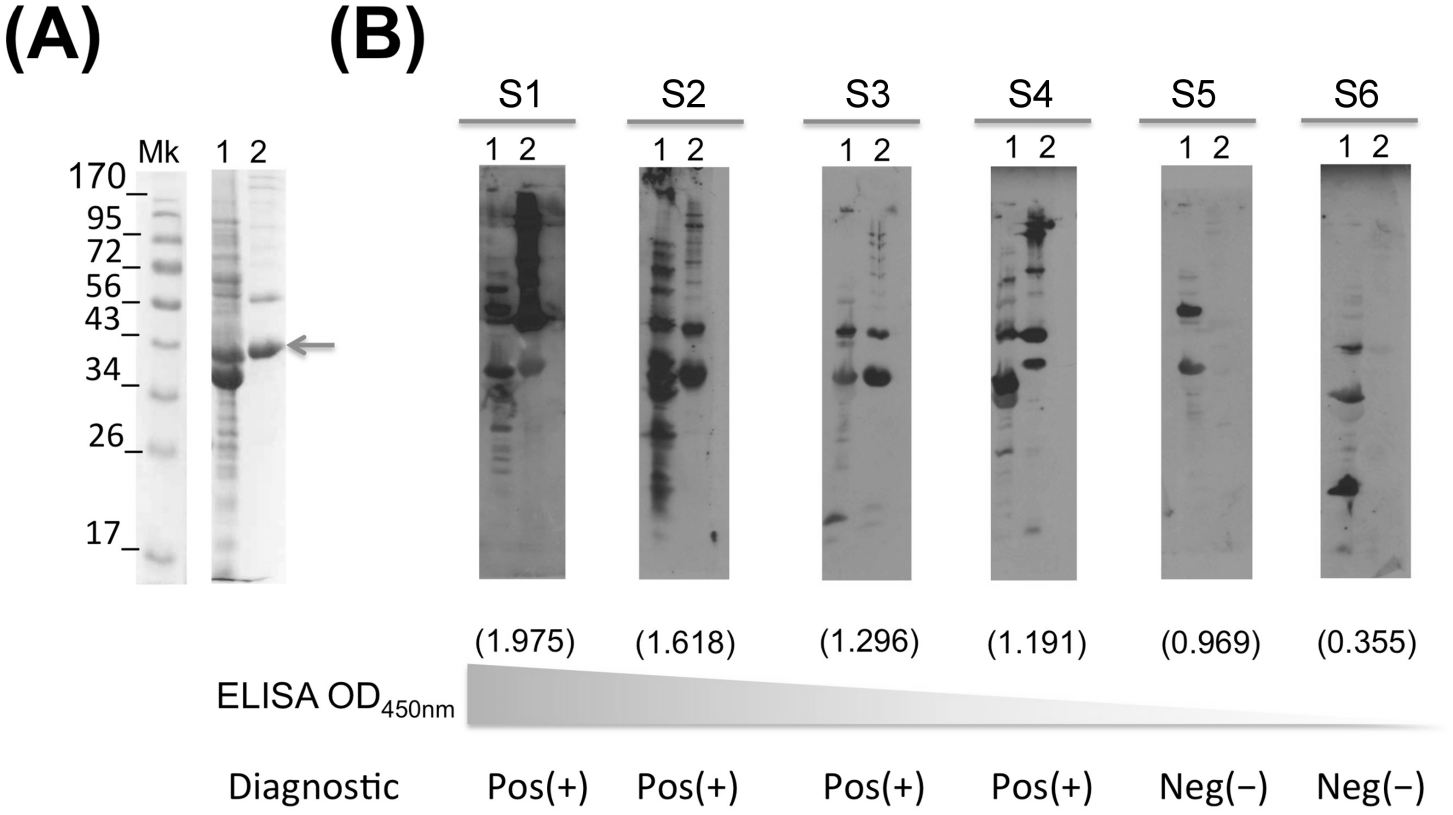
Immunoblot confirmation assay. *Borrelia turicatae* whole cell lysates were separated in 12% SDS-PAGE gels (line 1) together with rGlpQ (line 2) and stained with coomassie briliant blue (**A**). Western blots were run to confirm the ELISA sero-reactivity (**B**). S1 through S6 refers to representative positive and negative samples. Molecular weigt marker is indicated on the left side in kDa. The ELISA OD_450nm_ value is represented under each immunoblot in parenthesis. Pos (+) and Neg (-) describes the final result for each samples.

The 73 sero-reactive samples were further analyzed by western blot to confirm sero-reactivity to *B*. *turicatae* (Bt) whole cell lysates. As shown in Fig 3 sero-reactive samples to GlpQ were evaluated by western blot using both rGlpQ as well as *B*. *turicatae* whole cell lysates as antigens. Notice that sample 6 had a low ELISA value and a negative immunoblot. On the other hand, as the ELISA values increased the immunoblot assay reveals positive samples. It is worth noticing that some samples, such as number 5, have relatively high ELISA values with a negative immunoblot. When comparing the ELISA results with the immunoblot confirmation, we observed that out of the 73 (8.55%) sero-reactive samples (ELISA positive), only 17 (1.99%) were immunoblot positive.

**Fig 4:**
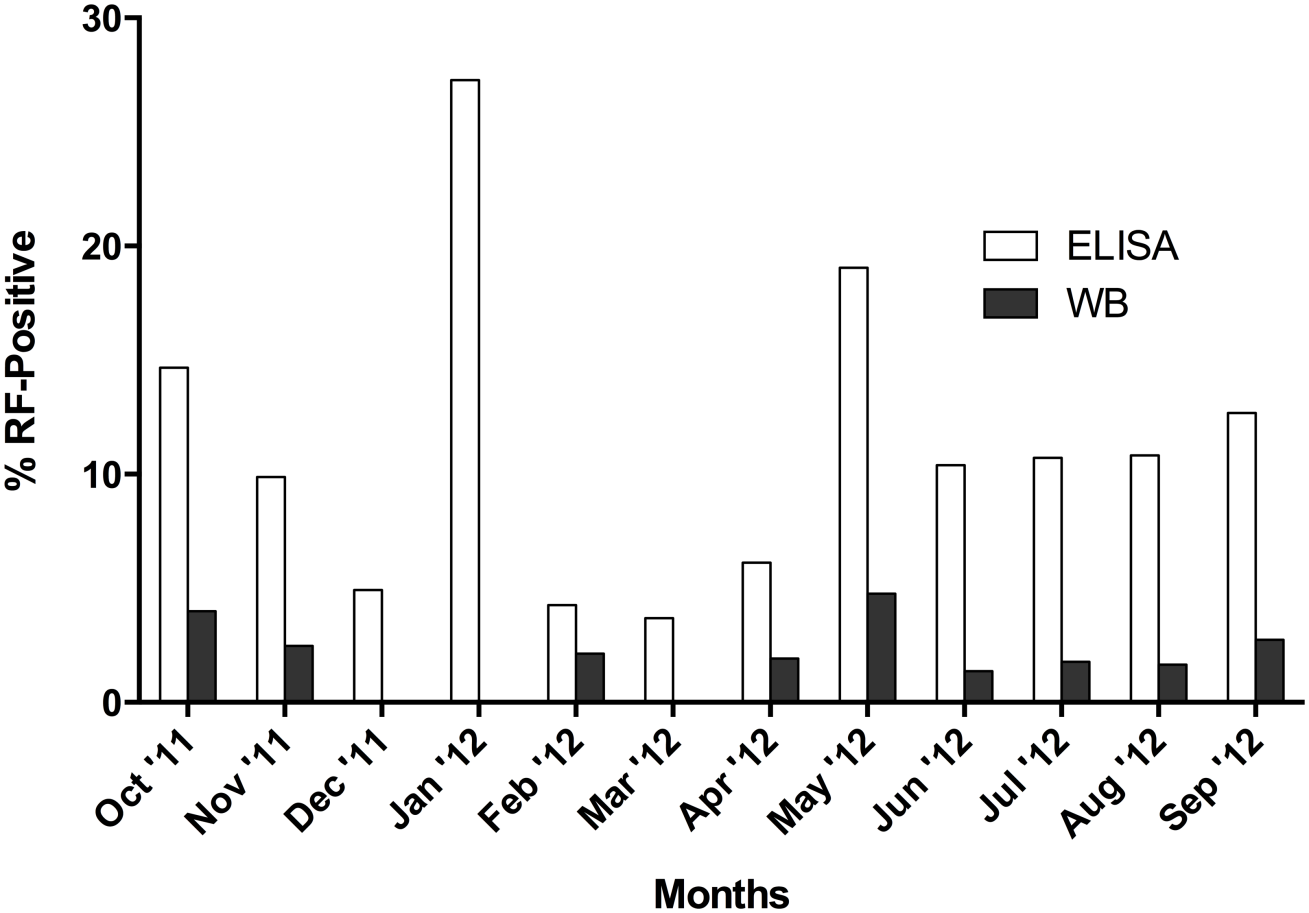
Percent sero-reactive canine samples by ELISA (white bars) and immunoblot (dark grey bars) during years 2011 and 2012. Percentage was calculated based on the total number of samples tested each month.

Since samples were collected from October 1^st^ 2011 until September 30^th^ 2012, we evaluated the temporal distribution of the tested specimens. As shown in Fig 4, the immunoblot assay reveals fewer reactive samples during winter months and spring (1 and 4, respectively) while most of the cases were detected during the fall (5 cases) and specially the summer (7 cases) months. Consequently 86.66% of the cases were detected from May-November, which correlate with the warmer months of the year.

**Fig 5:**
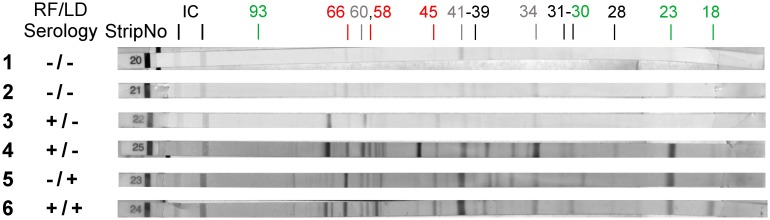
Lyme disease MarBlot^™^ Strip test was done to Tick-Borne Relapsing Fever (TBRF) sero-reactive samples. Strips 1 and 2 correspond with serum samples from dogs considered negative for both TBRF and Lyme disease (LD). Strips 3 and 4: correspond to dogs with positive serology for TBRF and negative serology for LD. Strip 5: corresponds to a dog with negative serology for TBRF and positive serology for LD. Strip 6: corresponds to a dog with positive serology for TBRF and LD. TBRF/LD Seology refers to the combined result of ELISA and Immunoblot assay for both diseases. Strip No. Refers to the provided numbers by the manufacturer. IC refers to the internal Controls on the strip. 93, 66, 60, 58, 45, 41, 39, 34, 31, 30, 28, 23 and 18 refers to the molecular weight in kDa of each *B*. *burgdorferi* antigen immobilized on the strip. Numbers in red refer to bands that lack specificity (cross-reactive) while the green numbers correspond with antigens used for the diagnostic of LD. Gray numbers correspond with antigens that have certain cross reactivity between TBRF and LD observed during this study.

In addition, to determine potential cross-reactivity between spirochetal bacteria from the same genus, all 73 Bt sero-reactive samples were tested to evaluate their reactivity to *Borrelia burgdorferi* (Bb) sensu stricto by IFA at TVMDL, and by ELISA and MarBlot^™^ at Dr. Esteve-Gasent’s laboratory [26]. As shown in Table 2, twenty-six samples (26/73, 35.62%) showed sero-reactivity to either Bb, Bt or both, Bb and Bt. In particular, 12.33% (9/73) of those samples were considered positive for Bb by IFA, ELISA and MarBlot^™^ test. Moreover, out of the 17 positive Bt samples, 15 were negative for Bb, but 2 samples were considered positive for both Bb and Bt by all tests run (Table 2). Fig 5 summarizes the Bb MarBlot^™^ test run in a representation of samples considered negative for both pathogens, positive for both, and positive for either one of the pathogens. In particular, the 2 double positive samples showed reactivity with a number of Bb antigens present on the MarBlot^™^ test as summarized in Table 3. Certain antigens, such as 60, 41 (flagella) and 34, were consistently present in all strips. Therefore, we have labeled them in gray in Fig 5, and on parenthesis in Table 3, denoting the possible cross-reactivity of such antigens between animals exposed to either Bb or Bt. In addition, antigen 66, 58 and 45 (labeled in red in Fig 5) were considered background bands, and also eliminated from the analysis. Thus, sero-reactivity to Bb was considered when tested serum reacted with antigens 93, 39, 31, 30, 28, 23 and 18. In order to evaluate potential presence of *Borrelia* in the double positive samples, a PCR reaction was done to DNA extracted from the serum obtained from those animals, obtaining negative results.

**Table 2:** Sero-reactive specimens to *Borrelia burgdorferi* and *Borrelia turicatae* by immunoblot assay.

		Average ELISA values	
Sero-reactivity	n (%)*	*B*. *burgdorferi* lysate	*B*. *turicatae* rGlpQ
*B*. *burgdorferi*	9 (12.33)	1.030 ± 0.369	1.480 ± 0.164
*B*. *turicatae*	15 (17.80)	0.769 ± 0.563	1.603 ± 0.226
*B*. *burgdorferi and B*. *turicatae*	2 (2.74)	1.732 ± 0.752	1.435 ± 0.330
Total	26 (35.62)		

* Out of 73 rGlpQ ELISA sero-reactive specimens.

**Table 3:** Reactivity of positive samples to *B*. *burgdorferi* MarBlot^™^ test and *B*. *turicatae* immunoblot test.

Sample	Bt ELISA values	Bb ELISA values	MarBlot^™^ bands*
1	1.669	2.263	93, (66), 60, (58), 41, 30, 23, 18
2	1.202	1.200	93, (66), 60, (58), (45), 41, 34, 31, 23, 18

* Numbers in parenthesis refer to cross-reactive bands.

**Fig 6:**
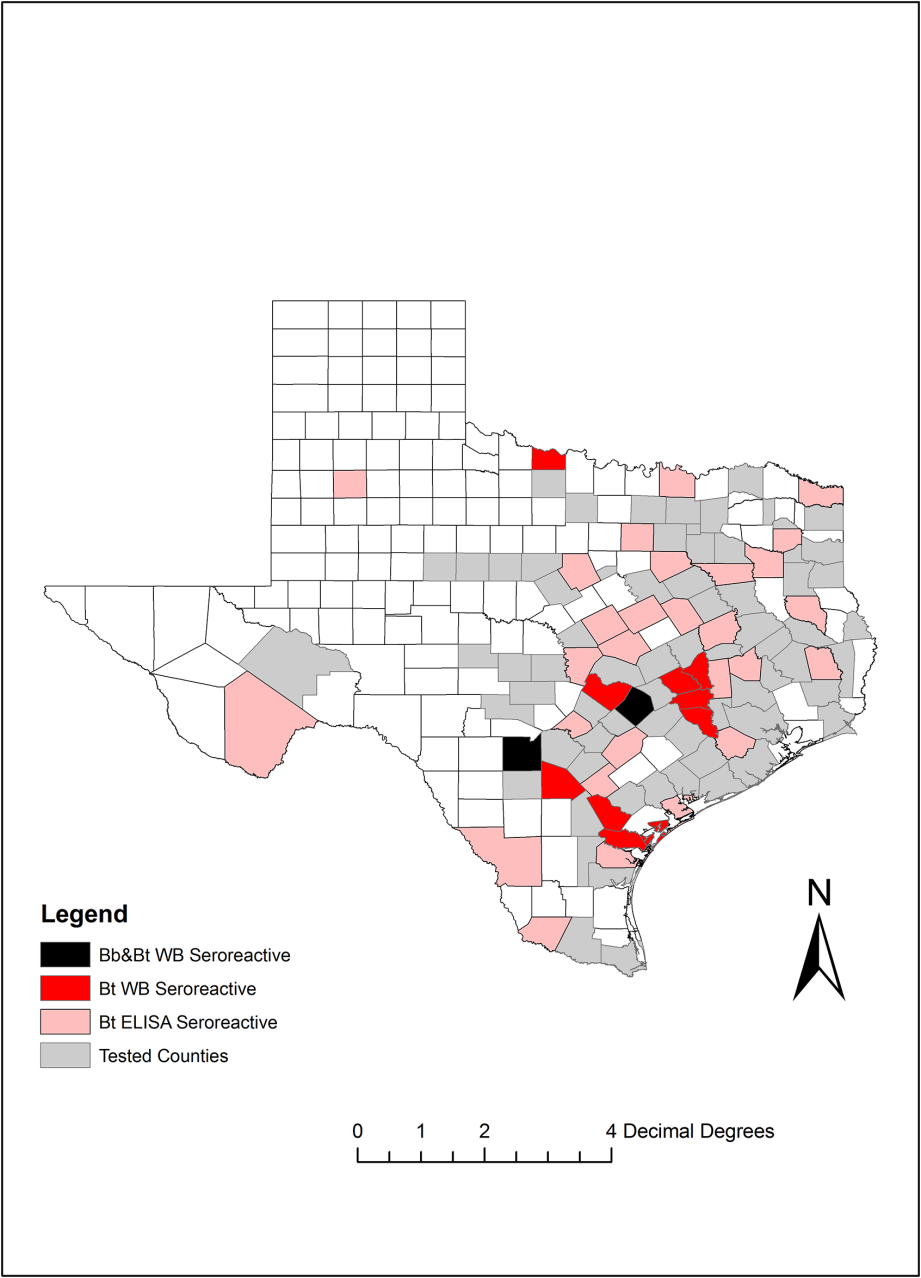
Geographic distribution of sero-positive samples as per immunoblot analysis. Localization by county of samples tested in this study. Map was generated using ArcGIS 9.0. Bb: *Borrelia burgdorferi*; Bt: *Borrelia turicatae*; WB: Western blot. Bb&Bt WB seroreactive refers to samples positive for both *B*. *burgdorferi* and *B*. *turicatae* by Western blotting.

Finally, Fig 6 shows the geographic distribution of the samples tested in the state of Texas. Most of the positive Bt samples were from counties in center-east Texas with the exception of Wichita county in northern Texas. In total, cases were reported from 12 different counties.

## Discussion

In this study, we have evaluated the presence of sero-reactive dogs to one of the causative agents of Tick-Borne Relapsing Fever (TBRF), *Borrelia turicatae*, in the state of Texas, U.S. At the time of manuscript composition, we collected a total of 878 serum samples from sick dogs (853 serum samples in Texas) that were submitted to the Texas A&M Veterinary Medical Diagnostic Laboratory, College Station, Texas (TVMDL) from October 2011 to September 2012. After evaluating the presence of antibodies specific to the *B*. *turicatae* GlpQ protein and whole cell lysates by both ELISA and Immunoblot assay we concluded that 1.99% of the canine samples tested demonstrated exposure to this pathogen. In addition, 2 samples (0.23%) were sero-reactive by all methods used for both *B*. *turicatae* and *B*. *burgdorferi* exposure. Even though human and canine TBRF cases have been sporadically reported in the literature, the current study is the first one evaluating the TBRF sero-prevalence in sick dogs in an endemic state throughout a 12 month period.

Currently, the diagnosis of canine TBRF is very limited, and based on screening of blood smears for the presence of spirochetes, or by running specific PCR for the *flaB* and *glpQ* genes [14, 15, 27]. Blood smear examination appears to be the most common method used in veterinary and human medicine, and it is considered the “gold standard” [3, 17, 22, 23, 28]. This methodology, while useful when animals are experiencing spirochetemia, clearly showing the spirochetes in blood, is also prone to numerous false negatives due to the temporality of the spirochetemia. Thus, a more specific test, such as serological diagnostics, or PCR during the febrile stages is preferred [14, 15, 22]. TBRF PCR analysis is not commercially available in any veterinary diagnostic laboratory; however, PCR has been performed in research laboratories to confirm clinical cases [3] and in reference laboratories in the diagnosis of African TBRF [22]. Previous work found that the *B*. *turicatae* recombinant protein Glycerophosphodiester phosphodiesterase (rGlpQ) could be used as antigen in an ELISA test in people [11, 18, 19]. This same team described another potential marker for the diagnostic of TBRF, the *Borrelia* immunogenic protein A, known as BipA [20, 21]. In those previous studies both antigens, rGlpQ and rBipA showed promising results in differentiating TBRF *Borrelia* infected animals from those infected with the Lyme disease (LD) *Borrelia*. Nevertheless, only rBipA has been evaluated as potential serological tests for TBRF in dogs [21] with promising results. Hence, our team approached the use of rGlpQ as a potential diagnostic tool for TBRF in Texas.

A total of 17 samples (1.99%) tested positive for TBRF. Unfortunately, due to significant background reaction, the use of GlpQ as a diagnostic marker was not very effective in discriminating dogs exposed to *B*. *turicatae*. The majority of serum samples that initially showed ELISA results above the set cut off value (Fig 2; 56 out of 73 samples), turned out to be negative once immunoblot assays were done using not only the recombinant GlpQ, but also *B*. *turicatae* whole cell lysates. This cross-reactivity was probably due to the exposure of dogs to other microorganisms that have GlpQ homologs (such as *Escherichia coli*, *Haemophilus influenza* and *Bacillus subtillis* among others [29]) with a percent homology ranging from 31 to 52%. On the other hand, *B*. *burgdorferi* does not encode for a *glpQ*, and therefore the cross-reactivity in the ELISA test cannot be due to the exposure to *B*. *burgdorferi*.

Interestingly, when running *B*. *burgdorferi* specific immunoblot assays we observed cross-reactivity of antigens 60kDa, 41kDa (Fla) and 31kDa (OspA) [30, 31] in samples that were considered *B*. *turicatae* positive but *B*. *burgdorferi* negative. This can be explained by the phylogenetic proximity of both species. Of the 17 positive samples to TBRF, 2 were sero-reactive to Bb. Following the discovery of these results and possible coinfections, the attending veterinarians were contacted and further clinical information was provided for one case. That case was an adult, intact male Beagle that presented for signs of neurologic disease, such as ataxia and running into objects. An in-house diagnostic test was negative for Lyme disease (4DX SNAP^™^ test, IDEXX Laboratories, Westbrook, ME). The dog had a history of tick exposure and thus, further tick-borne disease testing was pursued at a diagnostic laboratory (TVMDL), with a positive IFA for Lyme Disease, with a titer of ≥1:60. Thus, we suspect that most likely this patient was infected with *B*. *turicatae*, and the reactivity to *B*. *burgdorferi* might be due to cross-reactive bands. Therefore, further molecular tests need to be developed to improve the diagnosis of TBRF affecting both, humans and companion animals. This is even more relevant due to the sympatric circulation of *B burgdorferi* and *B*. *turicatae* in southern US.

In previous studies at the national level, this disease in humans was associated with summer exposures [2, 9, 24, 32]. When authors looked at the disease in patients that were exposed in Texas they observed a different seasonality compared with the rest of the country. In that study, human exposure was associated with rural dwelling and outdoor activities (i.e. cave exploring), with most of the cases diagnosed during the late fall and early winter months [32]. In our current study, we observed that most canine cases (12 out of 17) were detected in the summer (5 cases: June through September) and fall (7 cases: September through December) months, while fewer cases were observed in winter (1 case: December through March) and spring (4 cases: March through June). Our results are also in agreement with the recent findings by Piccione and collaborators [3]. Moreover, the dog cases tend to happen earlier in the year (June through December) compared with the observations made by Dworkin [32, 33] in human TBRF in the late 1990’s, where most of the human cases happened in November through January, suggesting a potential role of domestic dogs as sentinels for this disease. Thus, it is safe to consider TBRF a disease of risk during the warmer months of the year in all states considered endemic for the disease. In addition, part of the discrepancy in the seasonality of human versus canine TBRD could have been the diagnostic methodology used, PCR and serology *versus* microcopy. A lag phase is also observed between canine Lyme disease and human Lyme disease [34–37] in endemic areas of the disease. This observation has led to the use of dogs as sentinels for Lyme disease in such areas. Consequently, it will be reasonable to speculate that a similar effect could be observed in TBRF, where canine cases might appear earlier in the season due to the differences in human and canine behaviors that will impact exposure to infected ticks.

TBRF caused by the spirochetal pathogen *B*. *turicatae* has been described in Texas in multiple occasions in both humans and companion animals since the early 1980’s [3, 11, 14, 24, 25]. The reporting status of this disease keeps changing based on the number of cases detected in a particular state. In a recent study, Piccione and co-authors did a retrospective study of 5 cases of TBRF affecting dogs in the state of Texas [3], while Wilder and collaborators detected a sero-reactive human case in the same state [11]. Nevertheless, this is not a reportable disease in Texas, which makes it difficult to assess the current impact of this disease in both human and veterinary medicine.

Previous studies have shown the spatial distribution of both TBRF human cases [24] as well as the distribution of the tick vector *Ornithodoros spp*. [5]. Nevertheless, fewer studies have been done in order to provide a representation of the geographic distribution of TBRF in the country, and no studies have been published documenting the geographic distribution and seasonality of the disease in domestic dogs. For instance, Whitney and collaborators [14] reported 3 cases of canine TBRF in Texas. Two of the 3 cases were reported in the same county in which we have observed sero-reactive dogs in the current study. In addition, Piccione and collaborators, [3] also characterized 5 more cases in the state of Texas from locations closer to those presented in our current study. Thus, ours is the first report in which a geographic analysis of TBRF caused by *B*. *turicatae* is presented for the state of Texas, showing significant overlap with the reported presence of *O*. *turicata* in this state [5]. Nevertheless, there are no current surveys published in which the presence of infected *O*. *turicata* in Texas was evaluated, and thus, our report will stablish the first evidence of the distribution of *B*. *turicatae* in Texas.

There were several limitations of this study. We have used an opportunistic method for the sample collection using a restricted population of dogs that were suspected of infection with a tick-borne disease, and no apparently healthy dogs were evaluated. Thus, the claims on percent seropositive dogs can only be considered within dogs suspected of a tick-borne illness. Furthermore, clinical history, physical exam findings, and previous treatment protocols were not available to allow for correlation with testing results. In addition, besides Lyme disease, we do not know if the animals were diagnosed with any other infectious diseases. Due to retrospective nature of this study and the limited sample type (serum only), we could not confirm diagnoses by performing PCR on fresh samples. Nevertheless, we consider this study relevant since it was able to detect seropositive dogs to *B*. *turicatae* antigens in a state that has been consider endemic for the disease.

## Conclusions

Taken together, we have provided an analysis of the use of GlpQ as a potential marker for sero-diagnostics of TBRF in affected domestic canids. In addition, we have presented both the geographic and temporal distribution of canine TBRF caused by the spirochetal pathogen *B*. *turicatae* in dogs suspected of a tick-borne illness. As previously described, this disease was detected more frequently during the warmer months of the year. Based on the current findings, dogs seropositive for TBRF are present in Texas with very low incidence in the population studied (1.99%). Further studies are indicated to determine the best serologic test to detect TBRF in dogs. In addition, more research using larger canine populations and a randomized group of animals is necessary to better understand the real sero-prevalence of this disease in southern states in the U.S.

## Supporting information

S1 Table List of counties sampled in this study and number of samples tested in each county. (DOCX)

## References

[1] Cutler SJ. Relapsing Fever Borreliae: A Global Review. Clin Lab Med. 2015;35(4):847–65. doi: 10.1016/j.cll.2015.07.001 . 26593261 10.1016/j.cll.2015.07.001

[2] Forrester JD, Kjemtrup AM, Fritz CL, Marsden-Haug N, Nichols JB, Tengelsen LA, et al Tickborne relapsing fever—United States, 1990–2011. MMWR Morb Mortal Wkly Rep. 2015;64(3):58–60. . 25632952 PMC4584558

[3] Piccione J, Levine GJ, Duff CA, Kuhlman GM, Scott KD, Esteve-Gassent MD. Tick-Borne Relapsing Fever in Dogs. J Vet Intern Med. 2016 doi: 10.1111/jvim.14363 . 27353196 10.1111/jvim.14363 PMC5094544

[4] Gaither M, Schumacher M, Nieto N, Corrigan J, Murray H, Maurer M. Where Are the Ticks? Solving the Mystery of a Tickborne Relapsing Fever Outbreak at a Youth Camp. J Environ Health. 2016;78(8):8–11. . 27188066

[5] Donaldson TG, Perez de Leon AA, Li AY, Castro-Arellano I, Wozniak E, Boyle WK, et al Assessment of the Geographic Distribution of *Ornithodoros turicata* (Argasidae): Climate Variation and Host Diversity. PLoS Negl Trop Dis. 2016;10(2):e0004383 doi: 10.1371/journal.pntd.0004383 26829327 10.1371/journal.pntd.0004383 PMC4734830

[6] Boyle WK, Wilder HK, Lawrence AM, Lopez JE. Transmission dynamics of *Borrelia turicatae* from the arthropod vector. PLoS Negl Trop Dis. 2014;8(4):e2767 doi: 10.1371/journal.pntd.0002767 24699275 10.1371/journal.pntd.0002767 PMC3974661

[7] Lopez JE, Vinet-Oliphant H, Wilder HK, Brooks CP, Grasperge BJ, Morgan TW, et al Real-time monitoring of disease progression in rhesus macaques infected with *Borrelia turicatae* by tick bite. J Infect Dis. 2014;210(10):1639–48. doi: 10.1093/infdis/jiu306 24879799 10.1093/infdis/jiu306 PMC4326309

[8] Krishnavajhala A, Wilder HK, Boyle WK, Damania A, Thornton JA, Perez de Leon AA, et al Imaging of Borrelia turicatae Producing the Green Fluorescent Protein Reveals Persistent Colonization of the *Ornithodoros turicata* Midgut and Salivary Glands from Nymphal Acquisition through Transmission. App Environ Microbiol. 2017;83(5). doi: 10.1128/AEM.02503-16 27986725 10.1128/AEM.02503-16 PMC5311397

[9] Dworkin MS, Schwan TG, Anderson DE Jr., Borchardt SM. Tick-borne relapsing fever. Infect Dis Clin North Am. 2008;22(3):449–68, viii Epub 2008/08/30. doi: 10.1016/j.idc.2008.03.006 . 18755384 10.1016/j.idc.2008.03.006 PMC3725823

[10] Anderson JF. The natural history of ticks. Med Clin North Am. 2002;86(2):205–18. . 11982298 10.1016/s0025-7125(03)00083-x

[11] Wilder HK, Wozniak E, Huddleston E, Tata SR, Fitzkee NC, Lopez JE. Case report: A retrospective serological analysis indicating human exposure to tick-borne relapsing fever spirochetes in Texas. PLoS Negl Trop Dise. 2015;9(4):e0003617 doi: 10.1371/journal.pntd.0003617 25856342 10.1371/journal.pntd.0003617 PMC4391787

[12] Jones JM, Schumacher M, Peoples M, Souders N, Horn K, Fox L, et al Notes from the Field: Tickborne Relapsing Fever Outbreak at an Outdoor Education Camp—Arizona, 2014. MMWR Morb Mort Wkly Rep. 2015;64(23):651–2. . 26086637 PMC4584738

[13] Kelly AL, Raffel SJ, Fischer RJ, Bellinghausen M, Stevenson C, Schwan TG. First isolation of the relapsing fever spirochete, *Borrelia hermsii*, from a domestic dog. Ticks Tick Borne Dis. 2014;5(2):95–9. doi: 10.1016/j.ttbdis.2013.08.005 24252262 10.1016/j.ttbdis.2013.08.005 PMC3946889

[14] Whitney MS, Schwan TG, Sultemeier KB, McDonald PS, Brillhart MN. Spirochetemia caused by Borrelia turicatae infection in 3 dogs in Texas. Vet Clin Pathol 2007;36(2):212–6. . 17523100 10.1111/j.1939-165x.2007.tb00213.x

[15] Baneth G, Nachum-Biala Y, Halperin T, Hershko Y, Kleinerman G, Anug Y, et al *Borrelia persica* infection in dogs and cats: clinical manifestations, clinicopathological findings and genetic characterization. Parasit Vectors. 2016;9(1):244 Epub 2016/05/11. doi: 10.1186/s13071-016-1530-5 . 27160515 10.1186/s13071-016-1530-5 PMC4862127

[16] Breitschwerdt EB, Nicholson WL, Kiehl AR, Steers C, Meuten DJ, Levine JF. Natural infections with *Borrelia* spirochetes in two dogs from Florida. J Clin Microbiol. 1994;32(2):352–7. 8150943 10.1128/jcm.32.2.352-357.1994 PMC263035

[17] Halperin T, Orr N, Cohen R, Hasin T, Davidovitch N, Klement E, et al Detection of relapsing fever in human blood samples from Israel using PCR targeting the glycerophosphodiester phosphodiesterase (GlpQ) gene. Acta Tropica. 2006;98(2):189–95. Epub 2006/05/30. doi: 10.1016/j.actatropica.2006.04.004 . 16729949 10.1016/j.actatropica.2006.04.004

[18] Porcella SF, Raffel SJ, Schrumpf ME, Schriefer ME, Dennis DT, Schwan TG. Serodiagnosis of Louse-Borne relapsing fever with glycerophosphodiester phosphodiesterase (GlpQ) from *Borrelia recurrentis*. J Clin Microbiol. 2000;38(10):3561–71. Epub 2000/10/04. 11015364 10.1128/jcm.38.10.3561-3571.2000 PMC87437

[19] Schwan TG, Schrumpf ME, Hinnebusch BJ, Anderson DE Jr., Konkel ME. GlpQ: an antigen for serological discrimination between relapsing fever and Lyme borreliosis. J Clin Microbiol. 1996;34(10):2483–92. 8880505 10.1128/jcm.34.10.2483-2492.1996 PMC229300

[20] Lopez JE, Schrumpf ME, Nagarajan V, Raffel SJ, McCoy BN, Schwan TG. A novel surface antigen of relapsing fever spirochetes can discriminate between relapsing fever and Lyme borreliosis. Clin Vac Immunol. 2010;17(4):564–71. Epub 2010/02/12. doi: 10.1128/CVI.00518-09 20147497 10.1128/CVI.00518-09 PMC2849321

[21] Lopez JE, Wilder HK, Boyle W, Drumheller LB, Thornton JA, Willeford B, et al Sequence analysis and serological responses against *Borrelia turicatae* BipA, a putative species-specific antigen. PLoS Negl Trop Dis. 2013;7(9):e2454 Epub 2013/09/27. doi: 10.1371/journal.pntd.0002454 . 24069498 10.1371/journal.pntd.0002454 PMC3777926

[22] Fotso Fotso A, Drancourt M. Laboratory Diagnosis of Tick-Borne African Relapsing Fevers: Latest Developments. Frontiers Public Health. 2015;3:254 doi: 10.3389/fpubh.2015.00254 26618151 10.3389/fpubh.2015.00254 PMC4641162

[23] Fuchs I, Tarabin S, Kafka M. Relapsing Fever: Diagnosis Thanks to a Vigilant Hematology Laboratory. Vector Borne Zoonotic Dis. 2015;15(7):446–8. doi: 10.1089/vbz.2014.1764 . 26186517 10.1089/vbz.2014.1764

[24] Dworkin MS, Shoemaker PC, Fritz CL, Dowell ME, Anderson DE Jr., The epidemiology of tick-borne relapsing fever in the United States. Am J Trop Med Hyg. 2002;66(6):753–8. . 12224586 10.4269/ajtmh.2002.66.753

[25] Davis H, Vincent JM, Lynch J. Tick-borne relapsing fever caused by *Borrelia turicatae*. Pediatr Infect Dis J. 2002;21(7):703–5. . 12237608 10.1097/00006454-200207000-00020

[26] McGregor E, Boggess MM, Grover A, Rodgers S, Lupiani B, Esteve-Gassent MD. Lyme Disease in Clinically Sick Dogs in Southern US: Assessing Geographical and Seasonal distribution during year 2012–2011 in Texas. J Vet Sci Res. 2016;2(1). Epub May 12th 2016.

[27] Shirani D, Rakhshanpoor A, Cutler SJ, Ghazinezhad B, Naddaf SR. A case of canine borreliosis in Iran caused by *Borrelia persica*. Ticks Tick Borne Dis 2016;7(3):424–6. Epub 2016/01/19. doi: 10.1016/j.ttbdis.2015.12.020 . 26776536 10.1016/j.ttbdis.2015.12.020

[28] Wodecka B, Michalik J, Lane RS, Nowak-Chmura M, Wierzbicka A. Differential associations of *Borrelia species* with European badgers (*Meles meles*) and raccoon dogs (*Nyctereutes procyonoides*) in western Poland. Ticks Tick Borne Dis. 2016;7(5):1010–6. doi: 10.1016/j.ttbdis.2016.05.008 . 27263838 10.1016/j.ttbdis.2016.05.008

[29] Shang ES, Skare JT, Erdjument-Bromage H, Blanco DR, Tempst P, Miller JN, et al Sequence analysis and characterization of a 40-kilodalton *Borrelia hermsii* glycerophosphodiester phosphodiesterase homolog. J Bacteriol. 1997;179(7):2238–46. 9079909 10.1128/jb.179.7.2238-2246.1997 PMC178960

[30] Centers for Disease C, Prevention. Recommendations for test performance and interpretation from the Second National Conference on Serologic Diagnosis of Lyme Disease. MMWR Morb Mort Wkly Rep. 1995;44(31):590–1. . 7623762

[31] From the Centers for Disease Control and Prevention. Recommendations for test performance and interpretation from the Second National Conference on Serologic Diagnosis of Lyme Disease. JAMA. 1995;274(12):937 . 7674514

[32] Dworkin MS, Schwan TG, Anderson DE Jr. Tick-borne relapsing fever in North America. Med Clin North Am. 2002;86(2):417–33, viii–ix. . 11982310 10.1016/s0025-7125(03)00095-6

[33] Dworkin MS, Anderson DE Jr., Schwan TG, Shoemaker PC, Banerjee SN, Kassen BO, et al Tick-borne relapsing fever in the northwestern United States and southwestern Canada. Clin Infect Dis. 1998;26(1):122–31. Epub 1998/02/10. . 9455520 10.1086/516273

[34] Wagner B, Erb HN. Dogs and horses with antibodies to outer-surface protein C as on-time sentinels for ticks infected with *Borrelia burgdorferi* in New York State in 2011. Prev Vet Med. 2012;107(3–4):275–9. Epub 2012/07/31. doi: 10.1016/j.prevetmed.2012.07.002 . 22841496 10.1016/j.prevetmed.2012.07.002

[35] López-Alonso M. Pets as Sentinels of Human Exposure In: Nriagu JO, editor. Encyclopedia of Environmental Health. Burlington: Elsevier; 2011 p. 454–61.

[36] Schurer JM, Ndao M, Quewezance H, Elmore SA, Jenkins EJ. People, pets, and parasites: one health surveillance in southeastern Saskatchewan. Am J Trop Med Hyg. 2014;90(6):1184–90. Epub 2014/03/19. doi: 10.4269/ajtmh.13-0749 . 24639298 10.4269/ajtmh.13-0749 PMC4047752

[37] Smith FD, Ballantyne R, Morgan ER, Wall R. Estimating Lyme disease risk using pet dogs as sentinels. Comp Immunol Microbiol Infect Dis. 2012;35(2):163–7. doi: 10.1016/j.cimid.2011.12.009 . 22257866 10.1016/j.cimid.2011.12.009

