# Supplementary material for: Serological detection of Tick-Borne Relapsing Fever in Texan domestic dogs
Source: PLoS One. 2017 Dec 12;12(12):e0189786. doi: 10.1371/journal.pone.0189786 (PMC5726638; doi:10.1371/journal.pone.0189786)
Supplement: S1 Table — (DOCX) [file pone.0189786.s001.docx]

**Supplemental table 1**: list of counties sampled in this study and number of samples tested in each county.

| **County** | **N** | **County** | **N** | **County** | **N** |
| --- | --- | --- | --- | --- | --- |
| Anderson | 1 | Gonzales | 2 | Nacogdoches | 13 |
| Angelina | 3 | Grayson | 1 | Navarro | 3 |
| Aransas | 2 | Gregg | 4 | Nolan | 0 |
| Archer | 23 | Grimes | 5 | Nueces | 16 |
| Atascosa | 11 | Guadalupe | 1 | Panola | 1 |
| Austin | 27 | Hardin | 2 | Pecos | 1 |
| Bastrop | 28 | Harris | 20 | Polk | 1 |
| Bee | 7 | Harrison | 2 | Robertson | 1 |
| Bell | 21 | Hays | 3 | Rockwall | 2 |
| Bexar | 6 | Henderson | 10 | Rusk | 11 |
| Bowie | 9 | Hidalgo | 3 | Sabine | 1 |
| Brazoria | 3 | Hill | 1 | San Jacinto | 1 |
| Brazos | 82 | Houston | 4 | San Patricio | 2 |
| Brewster | 6 | Hunt | 7 | Smith | 28 |
| Burleson | 3 | Jack | 1 | Somervell | 3 |
| Burnet | 3 | Jackson | 3 | Starr | 1 |
| Caldwell | 1 | Jasper | 1 | Tarrant | 13 |
| Calhoun | 1 | Jefferson | 9 | Taylor | 3 |
| Callahan | 1 | Jim Wells | 3 | Travis | 18 |
| Cameron | 1 | Karnes | 7 | Trinity | 1 |
| Camp | 1 | Kaufman | 3 | Tyler | 1 |
| Cass | 5 | Kerr | 2 | Upshur | 3 |
| Collin | 2 | Kleberg | 1 | Van Zandt | 6 |
| Colorado | 1 | Lamar | 2 | Victoria | 3 |
| Comal | 3 | Lampasas | 1 | Walker | 15 |
| Comanche | 1 | Lee | 1 | Waller | 5 |
| Coryell | 19 | Leon | 2 | Washington | 17 |
| Dallas | 23 | Limestone | 4 | Webb | 23 |
| Denton | 1 | Live Oak | 13 | Wharton | 1 |
| Eastland | 2 | Llano | 4 | Wichita | 6 |
| Ellis | 8 | Lubbock | 5 | Williamson | 5 |
| Erath | 3 | Madison | 3 | Wilson | 1 |
| Fayette | 0 | Mason | 1 |  |  |
| Fort Bend | 11 | Matagorda | 1 |  |  |
| Franklin | 1 | McLennan | 23 |  |  |
| Freestone | 5 | Medina | 3 |  |  |
| Frio | 4 | Menard | 1 |  |  |
| Gillespie | 1 | Milam | 1 |  |  |
| Goliad | 4 | Montgomery | 6 |  |  |
